# Retrospective in silico evaluation of optimized preoperative planning for temporal bone surgery

**DOI:** 10.1007/s11548-020-02270-4

**Published:** 2020-10-11

**Authors:** Johannes Fauser, Simon Bohlender, Igor Stenin, Julia Kristin, Thomas Klenzner, Jörg Schipper, Anirban Mukhopadhyay

**Affiliations:** 1 Department of Computer Science, Technische Universität Darmstadt, Darmstadt, Germany; 2 Department of Oto-Rhino-Laryngology, Düsseldorf University Hospital, Düsseldorf, Germany

**Keywords:** Functional segmentation, 3D U-Net, Active shape models, Temporal bone, Robot-assisted surgery, Trajectory planning

## Abstract

**Purpose:**

Robot-assisted surgery at the temporal bone utilizing a flexible drilling unit would allow safer access to clinical targets such as the cochlea or the internal auditory canal by navigating along nonlinear trajectories. One key sub-step for clinical realization of such a procedure is automated preoperative surgical planning that incorporates both segmentation of risk structures and optimized trajectory planning.

**Methods:**

We automatically segment risk structures using 3D U-Nets with probabilistic active shape models. For nonlinear trajectory planning, we adapt bidirectional rapidly exploring random trees on Bézier Splines followed by sequential convex optimization. Functional evaluation, assessing segmentation quality based on the subsequent trajectory planning step, shows the suitability of our novel segmentation approach for this two-step preoperative pipeline.

**Results:**

Based on 24 data sets of the temporal bone, we perform a functional evaluation of preoperative surgical planning. Our experiments show that the automated segmentation provides safe and coherent surface models that can be used in collision detection during motion planning. The source code of the algorithms will be made publicly available.

**Conclusion:**

Optimized trajectory planning based on shape regularized segmentation leads to safe access canals for temporal bone surgery. Functional evaluation shows the promising results for both 3D U-Net and Bézier Spline trajectories.

## Introduction

**Fig. 1:**
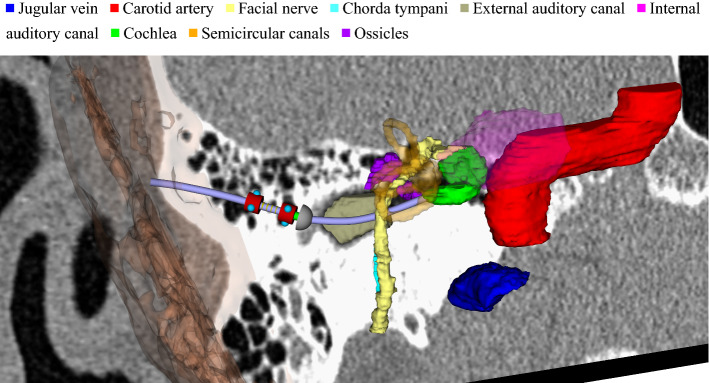
Robotic drilling of a nonlinear access canal through the temporal bone requires preoperative planning consisting of two steps: segmentation of risk structures within the temporal bone (white bone on the CT slice) and trajectory planning for a collision-free trajectory from the surface of the skull (transparent) to the clinical target (e.g., the cochlea)

Novel robot-assisted interventions have the potential to minimize patient trauma, reduce risk of infection or enable new surgical applications [2]. *At the temporal bone*, existing solutions focus on the drilling of linear access canals [4]. This paper addresses a novel nonlinear approach with the potential to increase safety as well as availability to more patients [6] (Fig. 1).

These robot-assisted surgeries require a two-step preoperative planning consisting of segmentation of risk structures and computation of nonlinear trajectories for the instruments. While surgeons currently rely on preoperative images and a mental 3D model of the anatomy, computational assistance for these new procedures will be fundamental due to the added complexity from both image processing and motion planning. Automation of tiresome and manually laborious tasks is therefore crucial for successful clinical implementation.

Dahrough et al. [4] provided a good review on existing systems and approaches for robotic temporal bone surgery. Solutions for entire preoperative planning in temporal bone surgery were presented by Noble et al. [13], Seitel et al. [5] and Gerber et al. [10] for linear approaches to the cochlea. More recently, nonlinear approaches to both cochlea and internal auditory canal were investigated by us employing nonlinear trajectories [8]. For the necessary segmentation of risk structures, approaches used either semiautomatic (Becker et al. [1]), traditional fully automatic methods (Noble et al. [12], Mangado et al. [11]) or deep learning approaches (Fauser et al. [8]).

So far, existing solutions mostly rely on semiautomatic segmentation and linear planning, while automatic approaches and nonlinear planning show insufficient precision leading to unsafe trajectories. We present a complete preoperative planning pipeline combining segmentation and nonlinear trajectory planning to a safe workflow. We then perform a thorough in silico evaluation of the whole approach on real patient data.

We propose a novel shape regularized 3D U-Nets approach for proper extraction of the tiny risk structures within the temporal bone. For subsequent computation of nonlinear trajectories, we adopt our sequential convex optimization (SCO) approach of [9] to generate locally optimal solutions. This two-step pipeline is evaluated in retrospective in silico experiments on 24 patients, where trajectories are computed on automatic segmentation results. Custom planning metrics assess robustness and safety of the process. These metrics include the effect that segmentation has on path planning and thus allow a more detailed analysis of the algorithms’ performance than image processing scores such as Dice alone. Quantitative evaluation of the complete pipeline shows that our segmentation approach combined with optimized Bézier Spline trajectories leads to collision-free access canals for two different applications: cochlear implantation and vestibular schwannoma removal.

## Objective

Robot-assisted temporal bone surgery uses image guidance based on a CT image, acquired shortly before surgery, to preoperatively determine a safe access canal to the clinical target. This could be the round window at the cochlea for a cochlear implantation or the internal auditory canal for vestibular schwannoma removal. An access canal is represented by a trajectory, constrained by the instrument’s maximum curvature $\kappa _\mathrm{max}\ge 0$ and a minimal safety distance to obstacles $d_\mathrm{min}>0$. It interpolates between a start configuration $q_I\in {\mathbb {R}}^3\times {\mathbb {S}}^2$ on the skull’s surface and a goal configurations $q_G\in {\mathbb {R}}^3\times {\mathbb {S}}^2$ at the target.

**Fig. 2:**
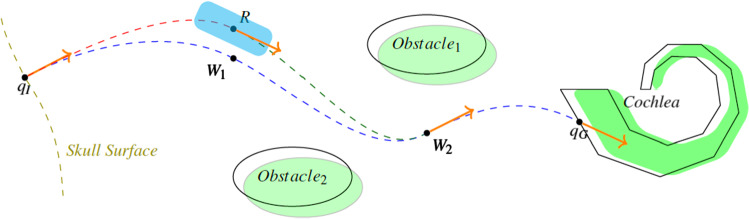
Sketch of surgical planning for an access canal from the skull’s surface to the cochlea. First, segmentation based on a preoperative CT image generates a surface representation (black) of risk structures (green objects). Then, motion planning computes collision-free trajectories from start $q_I$ to goal $q_G$. These trajectories are constrained by curvature, distance to obstacles and predefined initial and final configurations $q_I, q_G\in {\mathbb {R}}^3\times {\mathbb {S}}^2$, i.e., positions and direction. During intraoperative navigation, displacement of the robot *R* might necessitate replanning under the same constraints

A preoperative pipeline first segments risk structures of the temporal bone, in particular the internal and external auditory canal (IAC, EAC), the internal carotid artery (ICA) and jugular vein (JV), the ossicles (Oss), semicircular canals (SCC) and the cochlea (Coc) as well as facial nerve (FN) and chorda tympani (ChT). planning is necessary to guarantee patient safety. In a second step, a motion planning algorithm computes collision-free trajectories, where surface models extracted from segmentation are interpreted as obstacles.

Two key challenges appear: First, achieving topologically consistent segmentation, free from fragmented structures or inaccurate delineation, because this would lead to unsafe motion planning where successfully computed trajectories are in fact too close to obstacles. Second, motion planning for a collision-free nonlinear trajectory such that there is optimal clearance to risk structures. This enhances patient safety by increasing distance to obstacles and thus compensating for segmentation inaccuracy. The task of planning such a collision-free trajectory from the body’s surface to the clinical target is shown in Fig. 2.

**Fig. 3:**
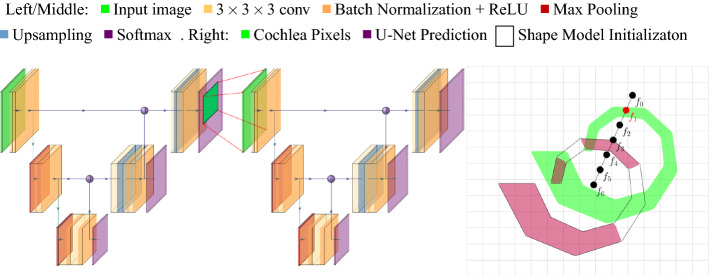
Our segmentation pipeline: Two 3D U-Nets of the same architecture predict an initial segmentation: the first (left) being applied on the input image, and the second (middle) on an extracted volume of interest. Right: Resulting fragmented surface meshes of this second prediction (purple) initialize probabilistic active shape models (black polygon) for each structure. These generate topologically consistent segmentations as final output

## Methods

We make a multi-step approach for automatic segmentation and nonlinear trajectory planning to solve this objective. A global 3D U-Net [3] coarsely segments the risk structures of the otobasis in a downsampled CT image. This gives an initial prediction of the nine risk structures. A second 3D U-Net of the same architecture predicts a finer segmentation on a bounding box computed from these results. To guarantee topologically consistent segmentation, we enforce shape constraints by regularization with probabilistic active shape models.

Clearance optimized trajectories are computed by a two-step approach. A bidirectional rapidly exploring random tree (Bi-RRT) on cubic Bézier Splines computes an initial solution. Because it will observe the characteristic stochastic twists and curves of a random sampling algorithm, we perform sequential convex optimization [9] to compute locally optimal solutions.

### Segmentation

We adopt shape regularized deep learning, which has shown great potential in combining state-of-the-art accuracy while enforcing topological constraints [8, 16]. Figure 3 shows the segmentation pipeline. Both U-Nets consist of five typical layers of repeated convolution, batch normalization, ReLU activation and pooling with respective upsampling and concatenation. We use combined Dice and weighted cross-entropy losses, which are also applied on intermediate layers following the approach of [18]. During training, Adam’s optimizer is used with a learning rate of 0.001 and two data sets are used during validation for early stopping. The course U-Net works on a $128^3$ cube created from a resampled version of the original CT image using cubic interpolation. The second U-Net is applied on the modified extracted bounding box of all structures but the ICA. In a typical CT scan of the otobasis, the remaining structures nicely align with the image axes, resulting in a major reduction of the original image’s size, and allow this second network to capture more detail. In particular, we only consider the largest connected component of each structure for the computation of this bounding box and create a volume of interest according to Algorithm 1, which leads to a volume of interest, that is square along axes *X* and *Y*, includes information about the Chorda Tympani despite resampling and is large enough to yield spatial information about all remaining risk structures. Shape regularization is achieved by applying probabilistic active shape model against the combined fine U-Net output, following the approach of [7]. These are initialized by nonrigid registration of the mean shapes onto the finer U-Net output and adapted to the image for several iterations. Unlike previous work [8], we achieve robust enough initial segmentations such that nonrigid registration does not collapse to irregular meshes.
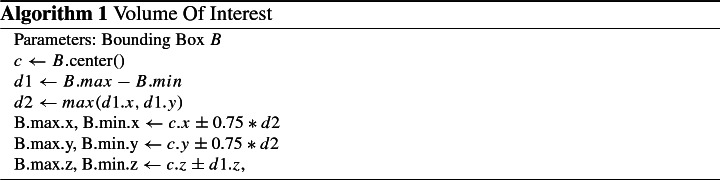


### Trajectory planning

**Fig. 4:**
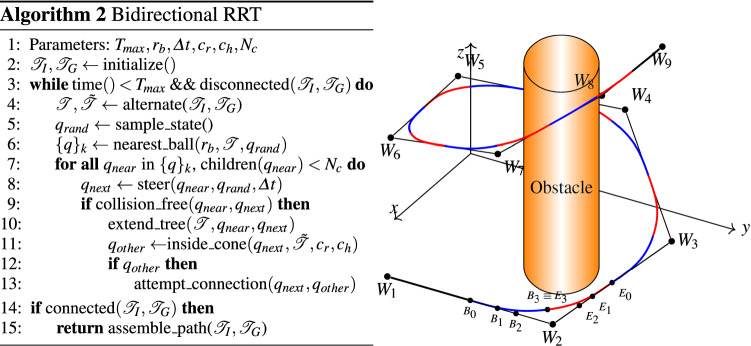
Left: Bi-RRT algorithm. Right: Resulting trajectory, consisting of waypoints $W1,\dots ,W9$ and implicitly defining cubic Bézier Splines (blue and red pairs). Each spline consists of two Bézier Spirals with control points $B_0\dots ,B_3$ and $E_0,\dots ,E_3$

We adopt sampling-based motion planning, which allows fast and robust initial planning [6, 14] in complex environments and sequential convex optimization as a stable solver for clearance optimization [9, 15]. Figure 4 with Algorithm 2 shows the proposed adaptation of a Bi-RRT on cubic Bézier Splines [6]. The search trees ${\mathscr {T}}_I,{\mathscr {T}}_G$ are initialized with the initial and goal states $q_I,q_G$. For a given time $T_\mathrm{max}$, the algorithm then tries to find a solution by alternately expanding ${\mathscr {T}}_I$ or ${\mathscr {T}}_G$. This is done by first sampling a random point $q_{rand}\in {\mathbb {R}}^3$ and computing the nearest neighbors in ${\mathscr {T}}$ around a ball with radius $r_b>0$. For each neighbor with less than $N_c$ child nodes, the steering function extends the tree along this trajectory using cubic Bézier Splines [17] with a step size of ${\varDelta } t$. If the trajectory is collision-free, the algorithm expands ${\mathscr {T}}$ and investigates possible connections to the other search tree. This is done using a cone with apex and direction defined by $q_{next}$ and with predefined parameters $c_r,c_h>0$ for radius and height. If successful, the result is an initial trajectory $T_I$ consisting of ${\mathscr {W}}\equiv \{W_i\}_i, 0\le i \le N_{\mathscr {W}},$ waypoints. Each triple $(W_{j-1}, W_j, W_{j+1}); 1\le j\le N_S \equiv N_{\mathscr {W}}-1$, implicitly defines a Bézier Spline $S_j$, a combination of two cubic Bézier Spirals, that respects the curvature constraint $\kappa _\mathrm{max}$. We refer the reader to [17] for a detailed description of the construction algorithm and proofs of smoothness and interpolation guarantees.

To reduce the natural stochastic twist of this initial RRT-solution, further optimization for smoothness and clearance to obstacles is necessary. We therefore define a constrained optimization objective over the set of waypoints ${\mathscr {W}}\subset {\mathbb {R}}^3$ that minimizes a cost function *f* while satisfying a set of $N_E$ equality and $N_I$ inequality constraints $h_i, g_j$, i.e.,

$$\begin{aligned} \displaystyle {\mathop {{{\,\mathrm{minimize}\,}}}\limits _{{\mathscr {W}}}}~~~&f({\mathscr {W}}) \\ \text {subject to}~~~&h_i({\mathscr {W}}) = 0,~~~i=0,\dots ,N_E \\&g_j({\mathscr {W}}) \le 0,~~~j=0,\dots ,N_I. \end{aligned}$$

**Fig. 5:**
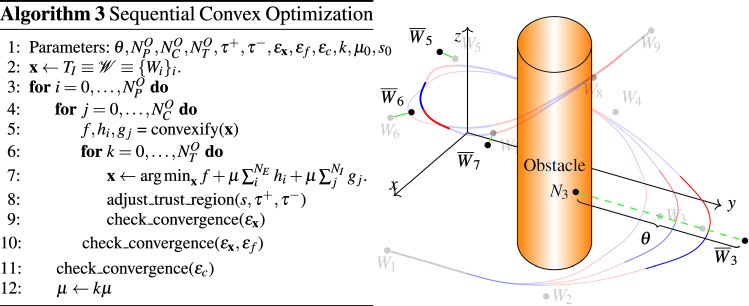
Left: Sequential convex optimization algorithm. Right: Schematic view of distance and curvature functions. At $(W_6$, the spline is straightened by moving ($W_5, W_6, W_7$) to new positions (${\overline{W}}_5, {\overline{W}}_6, {\overline{W}}_7$). At $W_3$, the distance cost is decreased by moving it further from the nearest neighbor $N_3$

Efficient numerical solvers require each of these functions to be linear or quadratic convex functions. In our case, these functions are, however, nonconvex and we thus consider an approximation rather than the original problem. By formulating adequate convex quadratic versions $f, h_i$ and $g_j$, convexifications, of the respective cost and constraint functions, we derive an approximation of our original problem that is suitable for numerical solvers. Algorithm 3 shows the proposed sequential convex optimization (SCO) approach of [15] for Bézier Spline trajectories: This iterative method repeatedly creates the convexified functions $f, h_i$ and $g_j$ based on the current solution $\mathbf{x }$ and makes progress on this approximated objective within a small trust region. Within each loop, tolerance checks on margins $\epsilon _f, \epsilon _\mathbf{x }, \epsilon _c$ for $f, \mathbf{x }$ and $h_i,g_j$, respectively, trigger adjustment of the trust region’s size, increase of penalty value $\mu$ or report of convergence. We refer the reader to [15] for a detailed description and show one iteration of the proposed clearance optimization method in Fig. 5 (right).

In particular, our cost function measures the quality of trajectories by a weighted sum of its length $f_{\varGamma }$ and distance to obstacles $f_{i,O}, 0\le i \le N_S$, i.e.,

$$\begin{aligned} f=\alpha _{\varGamma } f_{\varGamma } + \sum _i{\alpha _Of_{i,O}}, \end{aligned}$$

with $\alpha _{\varGamma }, \alpha _O\in {\mathbb {R}}^{0+}$. We approximate the length as

$$\begin{aligned} f_{\varGamma }=\sum _{i=0}^{N_{\mathscr {W}}-1}{\sum _{k=\{x,y,z\}}{|W_{i,k}-W_{i+1,k}|^2}}. \end{aligned}$$

Similar to [15], we measure distance to obstacles via linearized signed distances

$$\begin{aligned} \text {sd}_{SO}({{\mathbf {x}}}) = \text {sd}_{SO}({\mathbf {x_0}}) + {{{\mathbf {n}}}}({{\mathbf {x_0}}})^\top ({{{\mathbf {x}}}}-{{\mathbf {x_0}}}), \end{aligned}$$

where $\text {sd}_{SO}({\mathbf {x_0}})$ is the signed distance from a spline *S* to the nearest obstacle *O*, ${\mathbf {x_0}}\in O$ is a point on the surface and ${{\mathbf {n}}}$ the obstacle’s normal at ${\mathbf {x_0}}$. The point ${\mathbf {x_0}}$ stays fixed within an inner convex iteration sequence and is computed by a nearest neighbor search for ${{\mathbf {x}}}$. The weighted convexified clearance cost functions $f_{i,O}$ then try to match a distance threshold $\theta \in {\mathbb {R}}^+$ on the central waypoint $W_i$ of a spline $S_i$, i.e.,

$$\begin{aligned} f_{i,O} = \theta - \text {sd}_{S_iO}(W_{i}). \end{aligned}$$

We add constraints to guarantee the upper curvature bound $\kappa _\mathrm{max}$, the safety distance $d_\mathrm{min}$ and position and direction at $q_S,q_G$. To ensure that the upper bound $\kappa _\mathrm{max}$ on the curvature and the minimal distance $d_\mathrm{min}$ to obstacles stay valid during the optimization we introduce for each spline constraint functions $g_{i,\kappa }$ and $g_{i,O}, 0 \le i \le N_S$. Each curvature constraint $g_{i,\kappa }$ smooths its spline, if the upper bound $\kappa _\mathrm{max}$ is exceeded, by slightly translating the three corresponding waypoints. With $P_i=1/2(W_{i-1}+W_{i+1})$ and $Q_i=1/2(W_i+P_i)$, new waypoints ${\overline{W}}_{i-1}, {\overline{W}}_{i}, {\overline{W}}_{i+1}$ are given as

$$\begin{aligned} {\overline{W}}_{i-1}&= Q_i + (W_{i-1}-P_i),\\ {\overline{W}}_{i}&= \frac{1}{2}(W_i+Q_i),\\ {\overline{W}}_{i+1}&= Q_i + (W_{i+1}-P_i). \end{aligned}$$

A constraint $g_{i,\kappa }$ then penalizes the difference between the original positions and these translations, i.e.,

$$\begin{aligned} g_{i,\kappa } = \sum _{j=-1}^1\sum _{k=\{x,y,z\}}{|W_{i+j,k}-{\overline{W}}_{i+j,k}|^2}. \end{aligned}$$

The $g_{i,O}$ are defined like the distance cost functions via signed distances. Note, that we have to set $\theta>> d_\mathrm{min}$ to achieve significant improvement on clearance. Finally, we enforce that position and direction at start and goal stay the same by disallowing any changes in position of the first and last two waypoints.

We then use SCO [15] to solve for a locally optimal solution given the above costs and constraints.

## Experimental results

Data & Code Experiments were performed on 24 real temporal bone CT images of patients with an average resolution of $0.2\times 0.2\times 0.4$ mm$^3$. Corresponding label images were created by two fully trained clinicians, each annotating one half of the available images. Code of methods and experiments will be made publicly available on GitHub.^1^

**Table 1:** Parameter setup for motion planning algorithms

	$T_\mathrm{max}$	$r_b$	${\varDelta } t$	$c_r, c_h$	$N_c$			
Bi-RRT	0.1	1.0	4.0	5.0, 18.0	10			
	$N_P^O,N_C^O,N_T^O$	$\tau ^+,\tau ^-$	$\epsilon _\mathbf{x },\epsilon _f,\epsilon _c$	*k*	$\mu _0,s_0$	$\theta$	$\alpha _{\varGamma }$	$\alpha _O$
SCO	15, 50, 10	1.1, 0.9	$1e^{-4}, 1e^{-4}, 0.25$	1	0.5,0.1	5	1	10

**Table 2:** Segmentation performance in Dice and HD distances, *mean (standard deviation)*

Organ	Dice			HD		
	3D U-Net	ShapeReg	Ref [8]	3D U-Net	ShapeReg	Ref [8]
ICA	0.81 (0.05)	**0.87** (0.03)	0.84 (0.08)	3.32 (1.24)	**2.66** (0.94)	2.98 (1.57)
JV	0.68 (0.16)	**0.69** (0.16)	0.68 (0.14)	**4.22** (4.87)	4.45 (4.82)	4.60 (4.84)
FN	0.63 (0.09)	0.63 (0.20)	**0.69** (0.09)	4.18 (4.23)	**3.88** (4.00)	3.00 (2.84)
Coc	0.82 (0.04)	**0.87** (0.03)	0.85 (0.13)	1.36 (0.31)	**1.29** (0.51)	1.67 (1.99)
ChT	0.25 (0.17)	**0.39** (0.22)	0.36 (0.24)	**5.48** (9.00)	5.61 (8.52)	6.01 (9.83)
Oss	0.69 (0.13)	0.79 (0.13)	**0.82** (0.04)	**1.70** (0.97)	1.79 (0.82)	2.00 (1.28)
SSC	0.78 (0.06)	0.85 (0.03)	**0.84** (0.05)	**1.97** (2.69)	4.16 (5.01)	4.73 (4.88)
IAC	0.80 (0.09)	**0.84** (0.09)	0.83 (0.12)	**5.02** (4.77)	5.03 (4.74)	5.23 (5.16)
EAC	**0.81** (0.09)	0.80 (0.07)	**0.81** (0.08)	**3.60** (1.95)	3.89 (1.82)	4.12 (2.72)

Max/min values are in bold

Experiment Setup For each patient, we created surface models of the different structures from the expert annotations. In these environments, we manually placed start states $q_I$ at the skull’s surface and goal states $q_G$ at the round window of the cochlea as well as directly posterior and inferior to the IAC. We then defined three different scenarios for preoperative surgical planning with the same parameter setup as in [8]: one for a cochlear implantation (**Access**) with $\kappa _\mathrm{max} = 0.05, d_\mathrm{min} = 0.8$, and two for vestibular schwannoma removal (**SSC-Access**, through the superior SCC with $\kappa _\mathrm{max} = 0.05, d_\mathrm{min} = 1.5$, **RL-Access**, through the retrolabyrinthine region with $\kappa _\mathrm{max} = 0.05, d_\mathrm{min} = 2.0$). Table 1 lists the configurations for each of these scenarios.

We then performed a twofold cross-validation of the automated pipeline of Section 3 by dividing the 24 patient data sets into two equally sized subsets. Training of U-Nets and PASMs was performed on one set while testing was done on the respective other. After the segmentation step, three different sets of label images were available, expert annotations $L^{GT}$, U-Net segmentations $L^U$ and shape regularized versions $L^S$. From these images, we extracted surface models $S^{GT}, S^{U}, S^{S}$. The trajectory planning step was then executed three times, once on each set of surfaces models, leading to trajectories $T^{GT}, T^{U}, T^{S}$. We also compared against the shape regularized solution from [8] that uses a slice-by-slice approach, leading to $L^{2D}$, $S^{2D}, T^{2D}$.

We computed Dice and Hausdorff distances of $L^U$ and $L^S$ to measure segmentation performance independently. We then performed a functional evaluation of the whole pipeline using three metrics: The success rate $r_s$, quantifying the percental number of cases in which planning from surfaces $S^{GT},S^U,S^S$, was possible. The mean minimal distance to risk structures $r_d$ along trajectories $T^{GT}, T^{U}, T^{S}$. Finally, the failure rate $r_f$ for trajectories $T^{U}, T^{S}$, where the distance to risk structures of all paths was evaluated against $S^{GT}$ instead $S^U, S^S$, respectively. This rate quantifies the percental number a cases, where a trajectory planned on segmentation ($S^U, S^S$) violated the safety distance $d_\mathrm{min}$ when evaluated on $S^{GT}$ (the true position of risk structures). Consequently, $r_s$ measures the robustness of segmentation, thus detecting areas of oversegmentation. On the other hand, $r_d$ and $r_f$ quantify its safety by capturing areas of undersegmentation that lead to the computation of trajectories too close to risk structures.

**Table 3:** Results on planning metrics for each access canal and method

	Success rate			Mean safety distance ($d_\mathrm{min}$)			Failure rate ($d<d_\mathrm{min}$)		
	Coc	SSC	RL	Coc (0.8)	SSC (1.5)	RL (2.0)	Coc	SSC	RL
$T^{GT}$	**1**	**1**	**0.64**	1.02	1.91	2.87	–	–	–
$T^{U}$	**1**	0.88	0.48	**1.05**	2.05	**3.41**	**0.04**	0	0
$T^{S}$	0.96	**0.92**	**0.64**	1.03	1.99	2.88	**0.04**	0	0
$T^{2D}$	0.96	0.76	0.56	**1.05**	**2.24**	3.07	**0.04**	0	0

Max/min values are in bold

**Fig. 6:**
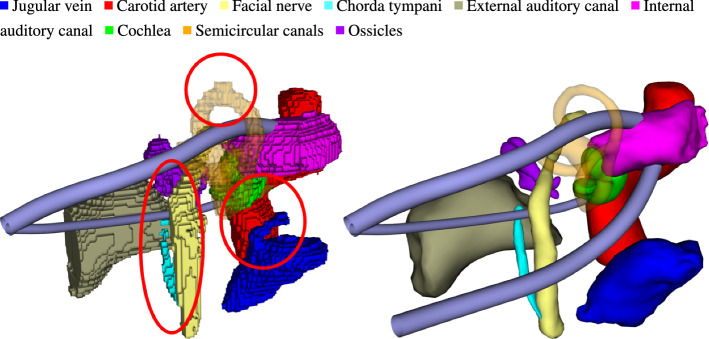
Segmented temporal bone anatomy from 3D U-Net (left) and regularization with probabilistic active shape models (right). The latter refines oversegmentation (e.g., SCC, FN), bridges small gaps (ChT) and removes artifacts from voxel-wise segmentation (JV), resulting in more robust trajectory planning

Results Dice and Hausdorff distances are shown in Table 2. Regularization improves Dice due to the shape model’s ability to bridge missing parts of a structure or ignore partial oversegmentation. This is noticeable especially for chorda tympani, Oss and SCC with absolute Dice improvements of about 14, 10 and $7\%$. We do not find major differences in Hausdorff distances (HD). Except for a single case of the SSC, where a mediocre U-Net result prevented proper initialization for the PASM model, these margins in HD are related to the open boundaries of the structures ([8]). We emphasize that due to our use of only the largest connected components from $L^U$, the 3D U-Net robustly detects the majority of individual structures. In comparison to the 2D approach of [8], we see a notable difference in performance for the FN. While the slice-by-slice approach clearly offers better initialization for this small tubular nerve, the advantage does not apply to segmentation of its side branch, the chorda tympani.

*Note 1* The 3D U-Net often outlined the clearly distinguishable structures such as cochlea or ossicles more precisely. While this might be favorable in applications such as electrode design, our shape regularized approach provides a more general and stable solution for the path planning step.

*Note 2* Our clinicians annotated some anatomical landmarks such as the bulb of the jugular vein slightly differently. However, both our neural networks and our active shape model regularization seem to cope with this issue well. In future work, we plan to investigate this further on larger data sets.

Planning metrics are given in Table 3 with a representative qualitative example in Fig. 6. The success rates are similar for all three methods in case of the Cochlea- and SSC-Access. The failing cases for shape regularization in both Cochlea- and SSC-Access we traced back to a bad initial segmentation $L^U$, resulting in inaccurate initialization of the PASM model for the SCC. This is also visible in the rather large HD for this organ. For the RL-Access, only our shape regularized approach achieves the same performance like $T^{GT}$. We found that the 3D U-Net fails to adequately delineate the high reaching jugular vein bulb (Fig. 6) and that general slight oversegmentation of the structures reduces the available free space. However, the rather low Dice of the JV comes again from the open boundaries at the inferior part of the structure ([8]). The mean safety distances show only minor differences. Although our SCO method provides locally optimal solutions, we hypothesize that the slight oversegmentation of 3D U-Net in contrast to the finer delineations of PASMs and expert annotations leads to higher safety distances.

**Fig. 7:**
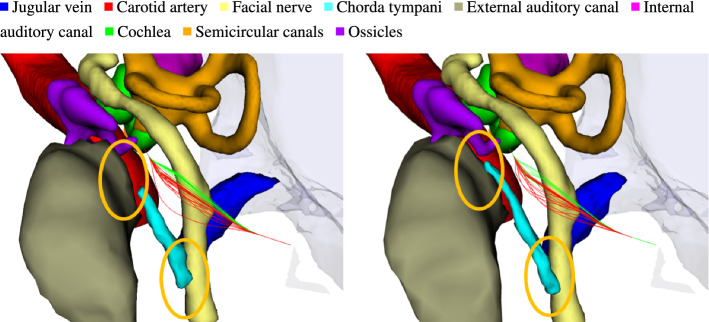
Comparison between shape regularized 3D U-Net (ours, right) and the slice-by-slice approach of [8] for the Cochlea-Access. The 3D U-Net provides preciser initialization of the active shape models, leading to robuster path planning. For the chorda tympani (cyan) in particular, it better captures its end points at the facial nerve and the tympanic cavity

We achieve safe access canals for both approaches to the internal auditory canal and a single failure case for the Cochlea-Access. While capturing of the whole chorda tympani was possible in the majority of cases, the 3D U-Net found only a small part at its superior end in the remaining cases. This then naturally applies to the shape regularized version and thus interferes with trajectory planning. Planning was still successful in most cases, because trajectories pass at the facial recess, but such segmentation is still not suitable for a reliable procedure. However, we found that in these cases the chorda tympani was still visually well distinguishable from neighboring tissue. This might thus be an issue coming from very low training data (10 cases) rather than a methodological problem. Finally, we note that failure rates for both Cochlea- and RL-Access significantly improved ($14\%, 10\%$) compared to former results of us [8]. The low failure rate for $T^{2D}$ indicates the effectiveness of convex optimization of trajectories. Comparing the success rates of $T^S$ and $T^{2D}$ shows that the 3D approach reduces oversegmentation and leads to better initialization of the PASMs, thus increasing robustness of the procedure (Fig. 7).

## Conclusion

We present a complete preoperative surgical planning pipeline for temporal bone surgery that computes nonlinear trajectories from the skull’s surface to the clinical target based on a CT image of the patient. The necessary segmentation of risk structures is automatically achieved by our novel approach using an initial prediction from 3D U-Nets and a refinement by probabilistic active shape models that regularizes the error prone pixel-wise predictions. Nonlinear trajectory planning follows a two-step approach [9] using bidirectional RRTs on cubic Bézier Splines that efficiently computes collision-free paths. A sequential convex optimization scheme further optimizes these trajectories regarding clearance to obstacles. We showed the suitability of our segmentation approach in a retrospective functional evaluation that includes both image processing and custom planning metrics.

Future work will evaluate this pipeline in the clinical work flow. Especially, the manual placement of start and goal states requires intuitive and ergonomic interaction. Furthermore, we plan to enrich the expert annotations with more label information, such as the brain, the temporal bone itself or the individual parts of the tympanic cavity. With a more detailed 3D representation of this cluttered anatomy and a suitable automatic segmentation method, this approach might be extendable to other clinical applications in this area. Additionally, a more diverse classification around the chorda tympani might also benefit the accuracy in this delicate region. Finally, we emphasize the 3D U-Net’s capability of completely segmenting the chorda tympani, indicating that with more available training data, shape regularized deep learning solutions promise fast and accurate segmentation of the complex temporal bone anatomy.

## Footnotes

1 https://github.com/MECLabTUDA/MUKNO.
